# Phase Inversion-Induced Porous Polybenzimidazole Fuel Cell Membranes: An Efficient Architecture for High-Temperature Water-Free Proton Transport

**DOI:** 10.3390/polym12071604

**Published:** 2020-07-19

**Authors:** Sangrae Lee, Ki-Ho Nam, Kwangwon Seo, Gunhwi Kim, Haksoo Han

**Affiliations:** Department of Chemical and Biomolecular Engineering, Yonsei University, 50 Yonsei-ro, Seodaemun-gu, Seoul 120-749, Korea; daniel2000alpha@gmail.com (S.L.); nam023497@gmail.com (K.-H.N.); kwseo@yonsei.ac.kr (K.S.); rlarjszzz@nate.com (G.K.)

**Keywords:** polybenzimidazole, porous membrane, thermal cross-linking, phosphoric acid, proton transport, high-temperature polymer electrolyte membrane fuel cells

## Abstract

To cope with the demand for cleaner alternative energy, polymer electrolyte membrane fuel cells (PEMFCs) have received significant research attention owing to their high-power density, high fuel efficiency, and low polluting by-product. However, the water requirement of these cells has necessitated research on systems that do not require water and/or use other mediums with higher boiling points. In this work, a highly porous *meta*-polybenzimidazole (*m*-PBI) membrane was fabricated through the non-solvent induced phase inversion technique and thermal cross-linking for high-temperature PEMFC (HT-PEMFC) applications. Standard non-thermally treated porous membranes are susceptible to phosphoric acid (PA) even at low concentrations and are unsuitable as polymer electrolyte membranes (PEMs). With the porous structure of *m*-PBI membranes, higher PA uptake and minimal swelling, which is controlled via cross-linking, was achieved. In addition, the membranes exhibited partial asymmetrical morphology and are directly applicable to fuel cell systems without any further modifications. Membranes with insufficient cross-linking resulted in an unstable performance in HT-PEMFC environments. By optimizing thermal treatment, a high-performance membrane with limited swelling and improved proton conductivity was achieved. Finally, the *m*-PBI membrane exhibited enhanced acid retention, proton conductivity, and fuel cell performance.

## 1. Introduction

As the demand for cleaner alternative energy sources increases, multiple fields of research have focused on potential renewable energy sources for the new generation. For applications that require instant power conversion from fuel, fuel cells are considered as a perfect candidate. Among the different types of fuel cells that have been developed, polymer electrolyte membrane fuel cells (PEMFCs) have captured the interest of several researchers due to their high-power density, high fuel efficiency, and low polluting by-product, compared to other fuel cells. Perfluorosulfonic acid polymer membranes, such as Nafion^®^, are widely used in PEMFCs as the polymer electrolyte membrane (PEM) for various applications owing to their favorable properties. However, such fuel cells are still associated with several problems that need to be resolved, such as low carbon monoxide tolerance, requirement of humidification equipment, expensive fluorinated polymers, and limited operating temperatures (below 100 °C), which leads to limited activity of expensive catalysts [1,2,3,4,5]. Particularly, the fundamental limiting aspect of perfluorosulfonic acid polymer is its requirement for water as the medium for proton transportation. Thus, systems which do not require water and/or use other mediums with higher boiling points are being researched.

High-temperature PEMFCs (HT-PEMFCs) can operate above 100 °C without humidification using acids such as phosphoric acid (PA) or ionic liquids as the medium. In comparison to conventional PEMFCs, they offer several advantages, including faster electrode kinetics, higher impurity tolerance, and a simplified systematic design [6,7,8,9,10]. Therefore, researchers have focused on developing PEMs with favorable characteristics for these HT-PEMFCs [11], such as polybenzimidazole (PBI). PBI possesses the necessary properties for HT-PEMFC application, such as high thermal and mechanical stabilities, low fuel cross-over, and resistance to chemical degradation [12,13]. However, it suffers from limitations such as lower proton conductivity than commercially available Nafion^®^. Thus, substantial efforts have been devoted toward improving the performance of PBI. Methods such as incorporating additives such as metal organic framework [14], graphene oxide [15,16], and other inorganic variants [17,18] have led to vast improvements in performance compared to base PBI and their various approaches are outlined in Table S1. In general, its proton conductivity can be directly improved by increasing its PA doping level. For this purpose, researchers have utilized different methods to create a porous membrane structure and substantially improve PA uptake, such as by varying the sizes of porogens, which are removed after membrane fabrication [19,20,21], and membrane formation through gelation [22,23]. The use of dibutyl phthalate as the porogen for PBI has been widely studied as it offers convenient control over porosity and viability [24,25]. However, using porogens can create large pores that can cause severe corrosion damage and carbon monoxide poisoning due to PA bleeding-out or chemical degradation of the membrane in the PBI/acid complexes.

Non-solvent induced phase separation (NIPS) is a widely studied method for the synthesis of a porous PBI membrane. It has been applied to various polymers in the fields of nanofiltration, separation, and reverse osmosis [26,27,28]. In addition, NIPS yields larger pore structures than other available methods. It is also easily controllable and suitable for PBI and other applications [29,30,31]. Porous membranes synthesized via NIPS exhibit excellent acid uptake, which directly correlates to their proton conductivity. To the best of our knowledge, membranes synthesized via this method for a high-temperature fuel cell system using PA have not been reported thus far [32,33,34]. Initial testing indicated the extremely high uptake of PA, resulting in the dissolution of the membrane at high temperatures, similar to that in a solvent [22,23,24,25,26,27,28,29,30,31,32,33,34,35]. To overcome this, the membranes are cross-linked to improve their stability and significantly reduce their solubility [36,37,38,39,40]. However, several approaches for cross-linking are performed during the film casting phase, which may interfere with the NIPS process. Thus, the ideal thermal cross-linking method an appropriate approach to overcome membrane dissolution as it can be conducted after membrane fabrication [41,42,43].

In this work, we synthesize porous *meta*-PBI (*m*-PBI) membranes via NIPS. The *m*-PBI was chosen over other candidates such as p-PBI, as their inherently reduced solubility hindered the porous membrane fabrication process. Chemical and morphological analyses of the synthesized membranes were conducted using Fourier transform infrared spectroscopy (FT-IR), thermogravimetric analysis (TGA), and scanning electron microscopy (SEM), in order to optimize and evaluate cross-linking and performance through interactions with PA. Moreover, PA doping and swelling were characterized via doping level testing. The performance of the membrane for application in HT-PEMFCs was evaluated based on proton conductivity using an Impedance Analyzer. Furthermore, membrane electrode assembly (MEA) was performed to determine the single cell performance of the membrane.

## 2. Experimental

### 2.1. Materials

For the synthesis of *m*-PBI, isophthalic acid (IPA) and polyphosphoric acid (PPA, 115%) were purchased from Sigma Aldrich (St. Louis, MI, USA). Furthermore, 3,3′-diaminobenzidine (DAB) was purchased from TCI Chemicals (Tokyo, Japan). Sodium carbonate (Na_2_CO_3_), dimethylacetamide (DMAc), acetone, and isopropyl alcohol were purchased from DUKSAN Chemical (Seoul, South Korea). Deionized (DI) water was obtained from a Milli-Q Ultrapure water purification system. For the acid doping of the membranes and the MEA fabrication, PA (85 wt% in H_2_O) was purchased from Sigma Aldrich. For MEA fabrication, polytetrafluoroethylene (PTFE, 60 wt% in H_2_O) as the binder was purchased from Sigma Aldrich, and Pt/C (40 wt% on carbon) was purchased from Alfa Aesar (Ward Hill, MA, USA). A carbon cloth (HT1400W, BASF) was used as the gas diffusion layer. All reagents used in this work were used as received without any further purifications.

### 2.2. Synthesis of m-PBI

**Figure 1:**
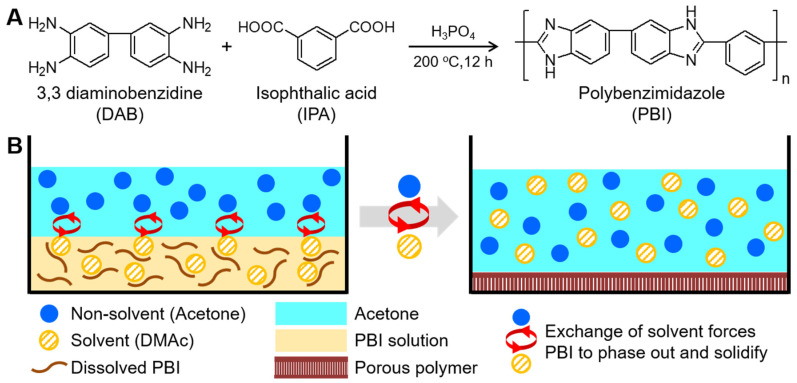
(**A**) Synthesis of *meta*-polybenzimidazole (*m*-PBI) and (**B**) the non-solvent induced phase separation mechanism to achieve porosity.

The *m*-PBI was synthesized via the polycondensation method, which was reported by Kumbharkar et al. [35], with minor adjustments. The overall reaction is depicted in Figure 1A. In a three-neck flask with a mechanical stirrer, thermometer, and nitrogen inlet, DAB (2.165 g) was dissolved in PPA (75 g) at 120 °C. IPA (1.678 g) was then added to this solution, and the temperature was increased to 200 °C. The solution was stirred for 12 h, during which its viscosity gradually increased with the synthesis time. The solution was subsequently poured into DI water and was consecutively washed until it was neutral. Thereafter, the precipitates were kept in 10% aqueous Na_2_CO_3_ solution for 12 h to remove any residual PPA within the polymer matrix. Finally, the precipitates were collected and dried in vacuum at 80 °C for three days to remove any traces of water.

### 2.3. Preparation of Highly Porous m-PBI Membranes

The synthesized *m*-PBI powder was added to create a 10 wt% solution in DMAc, and the solution was stirred at 60 °C. After achieving a homogeneous solution, it was uniformly spread onto a glass plate using a spin coater. The cast solution was placed in acetone for 3 h to ensure maximum solvent exchange (Figure 1B). Subsequently, it was placed in vacuum for 8 h at 80 °C to remove the acetone and residual solvent. It was then washed with DI water, peeled, and dried overnight to remove the residual water. This sample was heated to 350 °C in a ceramic oven for different amounts of time (up to 3 h) before cooling to 25 °C. The samples were then labeled *m*-PBI350-x, where x denotes the duration of heating (h) at 350 °C. As a reference, a non-porous *m*-PBI film was produced using DMAc without the phase inversion process and spread on a glass plate; the solvent was removed by placing this sample under vacuum at 80 °C for 8 h. All samples for the electrochemical analyses were doped with 85 wt% PA for 3 days at 60 °C to achieve the maximum uptake.

### 2.4. Fabrication of PEM Fuel Cell MEA

The catalyst solution was prepared by adding the desired amount of Pt/C (46.7 wt% Pt, Tanaka Kikinzoku Kogyo K.K, Chiyoda City, Japan) and PFTE in a water/isopropyl alcohol solution with a ratio of 4:1 w/w. To maintain good dispersion, the concentration of the catalyst/binder should be ~1%. The solution was then sonicated for 30 min to ensure even dispersion and sprayed onto gas diffusion layers using a spray gun, to create a gas diffusion electrode (GDE). The catalyst loading was 1.5 mg_Pt_ cm^−2^ for both sides. Thereafter, the GDE was annealed at 350 °C for 10 min. The prepared GDEs were used to sandwich the PA-doped membrane fitted with a gasket in a single fuel cell at 75 N m for MEA testing.

### 2.5. Characterization

The synthesis of the membrane was confirmed via FT-IR (VERTEX 70v, Bruker, Billerica, MA, USA), and its thermal stability was measured using TGA (Q50, TA Instrument, New Castle, DE, USA). The structure of the membrane was observed using SEM (JEOL-6701F, JEOL, Akishima, Japan) after Pt sputtering.

The porosity of the membranes was measured by method previously reported [44], where the membranes were immersed in *n*-butanol for 1 h and the weight of before and after immersing was recorded. The porosity was calculated via the following equation.

(1) $$P\%\;=\frac{M_{B}/\rho_{B}}{M_{\mathrm{PBI}}/\rho_{PBI}+M_{B}/\rho_{B}}$$

where P% is porosity of the membrane, *M*_PBI_ the mass of *m*-PBI membrane, *M*_B_ the mass of absorbed *n*-butanol, *ρ*_PBI_ the density of the membrane and *ρ*_B_ the density of *n*-butanol.

The membranes were dissolved in the selected solvent and heated at 80 °C in DMAc and at 160 °C in PA for a specific amount of time. The samples were washed with water and dried to remove the absorbed solvent. The solubility of the membranes was then determined by obtaining their gel content, as follows:

(2) $$\mathrm{Gel}\;\mathrm{content}\;\left(\mathrm{wt}\%\right)=100*\left(\frac{w_{2}}{w_{1}}\right)$$

where *w*_1_ is the initial weight, and *w*_2_ is weight after dissolution.

To calculate the doping level of the membranes, the amount of doped PA was estimated by weighing the membrane before and after doping. The initial weight of the membrane (*W_dry_*) was recorded by drying in a vacuum oven at 80 °C. The membrane was then removed and weighed after wiping off excess PA (*W_wet_*). The doping level was calculated by

(3) $$\mathrm{ADL}\;=\frac{\left(W_{wet}-W_{dry}\right)/M_{PA}}{W_{dry}/M_{PBI}}$$

where ADL is the acid doping level, *M*_PA_ and *M*_PBI_ denote the molecular weights of PA and *m*-PBI repeat units, respectively.

Swelling ratio of the samples was also noted and calculated by

(4) $$\mathrm{Swelling}\;\mathrm{ratio}\;\left(\%\right)=100*\frac{A_{wet}T_{wet}-A_{dry}T_{dry}}{A_{dry}T_{dry}}$$

where *A* is the area of the membrane, and *T* is its thickness; *wet* and *dry* indicate the undoped and doped states, respectively.

The PA retention ability of the composite film was also investigated. An acid leaching test was performed, similar to that reported by Quartarone et al. [45]. The migration stability of the acid in the composite membranes was characterized by the PA weight loss (*R*) in the membrane, which was obtained under a water vapor condition by hanging the membrane above boiling water for a certain period to allow the water drops to coalesce on the membrane surface. These membranes were then periodically removed and dried in order determine their weight (*W_i_*). The *R* of PA from the membrane was calculated by

(5) $$R\;=\frac{W_{0}-W_{i}}{W_{PA}}\times 100\%$$

where W_0_ is the initial doped weight of the membrane, and *W_PA_* is the weight of the doped PA inside the membrane, calculated using Equation (5).

The proton conductivity of the PA-doped composite membranes was measured using electrochemical impedance spectroscopy with a frequency range of 0.1 Hz to 100 kHz. The four-probe test method was carried out using a potentiostat (Reference 600, Gamry) and a conductivity cell without humidification to calculate the resistance from Nyquist plot. Subsequently, the conductivity was calculated as follows:

(6) $$\mathrm{Proton}\;\mathrm{Condcutivity}\;\left(S/cm\right)=\frac{d}{\Omega A}$$

where *d* is the distance between two electrodes, *Ω* is the resistance of the membrane, and *A* is the cross-sectional area of the measured membrane. For temperature dependence of proton conductivity, membrane was kept at 160 °C prior to measurement to remove traces of water for more reliable measurement.

Based on linear regression, the activation energy was calculated using the proton conductivity of the membranes to plot the Arrhenius equation:

(7) $$\sigma \;=\sigma_{0}\exp\left[\frac{-E_{a}}{\mathrm{RT}}\right]$$

where σ is the proton conductivity (S cm^−1^), σ_0_ is the pre-exponential factor (S K^−1^ cm^−1^), E_a_ is activation energy (kJ mol^−1^), *R* is the ideal gas constant (J mol^−1^ K^−1^), and *T* is the absolute temperature expressed as 1000/K.

MEA was evaluated at 160 °C on porous *m*-PBI350-2 and non-porous *m*-PBI membranes in a single cell using a cell station (SMART2, WonATech, Seoul, South Korea). The active area was 1 cm^2^, Pt loading was 1.5 mg cm^−2^, and the flow rates of H_2_ and O_2_ were 100 ccm. They were activated with a current load of 200 mA cm^−2^ prior to the measurement.

## 3. Results and Discussion

**Figure 2:**
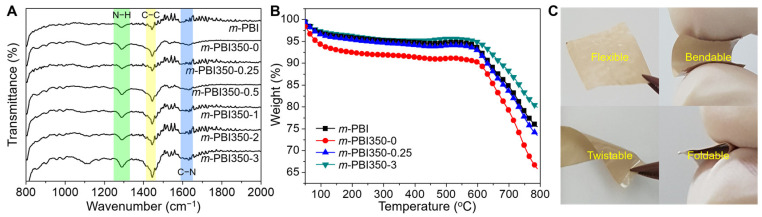
(**A**) Fourier transform infrared spectra and (**B**) thermogravimetric analysis of non-porous *m*-PBI and *m*-PBI350-x membranes. (**C**) Demonstration of the robustness of the freestanding *m*-PBI350-2.

FT-IR was used to observe the synthesis of the *m*-PBI membranes and the chemical changes caused by the phase inversion and thermal curing. Figure 2A shows the FT-IR spectra of the prepared membranes. There are no noticeable peaks around the 1700 cm^−1^ region, which corresponds to the C=O stretch of carbonyl groups; thus, almost all of these groups participated in the formation of benzimidazole rings. The absorption of the N−H group and aromatic C−C stretching was observed at 1280 cm^−1^ and 1440 cm^−1^, respectively. C=C/C=N stretching was observed at 1610 cm^−1^. These results are in good agreement with existing literature on the successful synthesis of *m*-PBI membranes [46]. In addition, there are no significant differences between the species, confirming that the NIPS process was not hampered by their chemical structures.

As *m*-PBI is known for its excellent thermal stability, the effect of the NIPS process and thermal curing on this property was studied via TGA, as shown in Figure 2B. Non-porous *m*-PBI are relatively stable up to 500 °C, which is significantly higher than their operating temperatures in fuel cells. The initial weight loss, which begins at approximately 100 °C, is attributed to the release of absorbed water and/or any residual solvent [8,9]. At higher temperatures of approximately 600 °C, the degradation of the main *m*-PBI backbone can be observed, showing typical temperature profile of aromatic PBI [47]. There are no other noticeable peaks, and the thermal treatment did not alter the thermal profile of the membranes. The phased inversion-induced freestanding *m*-PBI membranes could also maintain their original shapes without cracking after releasing the applied bending, twisting, and folding forces (Figure 2C).

**Figure 3:**
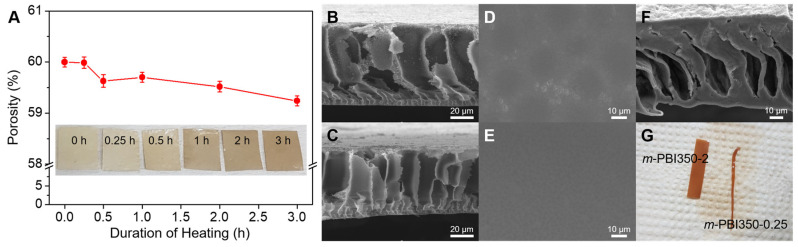
(**A**) *n*-Butanol adsorption porosity (%) values of *m*-PBI350-x membranes with respect to duration of heating time at 350 °C (inset: digital camera photographs of *m*-PBI350-x membranes). Scanning electron microscopy images of the cross-section of (**B**) *m*-PBI350-0 and (**C**) *m*-PBI350-3; and the membrane surface of (**D**) *m*-PBI350-3 glass side and (**E**) air side. (**F**) Cross-section of *m*-PBI350-0.25 and (**G**) image of the membrane after acid removal.

In order to determine the porosity of the membranes, an *n*-butanol adsorption test was carried out (Figure 3A). It was seen that the membranes synthesized via NIPS method all had a porosity of around 60%. The porosity also decreased with increased thermal treatment, possibly due to densification from crosslinking. However, the degree in which the thermal treatment affects the porosity is negligible and should not affect the acid uptake properties. The surfaces of the membranes were subjected to SEM, and their cross sections were obtained to observe the morphological structures and successful phase inversion of *m*-PBI membranes. As shown in Figure 3A–F, a highly porous structure is achieved for all phase-inversed membranes with the distribution of both large and small pores. The middle section exhibits pillar-like macroporous structures. On magnifying the walls, highly porous layers are observed. In addition, filling up all these pores may reduce the amount of gas cross-over, which is a major concern of MEA tests in HT-PEMFCs as it could lead to severe gas cross-over that decreases its performance and durability or causes immediate failure upon initial testing [48]. As observed in the cross-section, the surface of the membrane does not exhibit any large pore openings. The surface images indicate the presence of underlying closed pores, which is confirmed by magnifying the surfaces with a negligible amount of pores (Figure 3D–E). This is beneficial for a fuel cell membrane as it reduces the gas cross-over. Moreover, an asymmetric membrane is derived via the NIPS process. There are no significant differences in the morphology of the neat and thermally cured porous samples. This was confirmed from porosity measurement, where it was seen that porosity somewhat decreased with increased thermal treatment, and the variances were negligible. During the acid doping test, partial dissolution was observed for the samples with low curing time at elevated temperatures (>80 °C), where the membrane became visibly semi-transparent and some dopant leached out upon heating. This suggests that the pores collapsed due to the partial dissolution and insufficient cross-linking of the membrane (Figure 3F). The cross-section of the membrane was investigated after the removal of PA. The images confirm the collapse of the pores. In addition, the membrane was permanently deformed and became extremely brittle after drying, making it unsuitable for HT-PEMFCs. Thus, a sufficient curing time is necessary for the HT-PEMFC environment.

**Figure 4:**
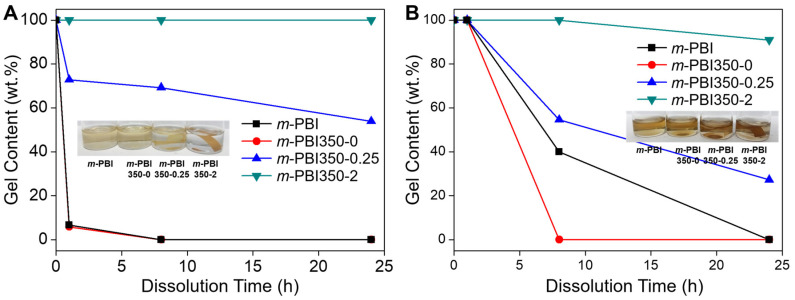
Solubility with respect to time in (**A**) dimethylacetamide (DMAc) (80 °C) and (**B**) phosphoric acid (PA) (160 °C). The inset images were captured after testing for 24 h.

To determine the decreased solubility from the thermal cross-linking of the phase-inversed membrane for HT-PEMFCs, the solubility of the membranes was measured in DMAc at 80 °C and PA at 160 °C. Previous studies indicated a drastic reduction in the solubility of membranes thermally treated at 350 °C in DMAc and PA [41,49]. From Figure 3F,G, it is evident that there is insufficient thermal treatment of the membranes, resulting in partial dissolution and the collapse of pores. In DMAc, both *m*-PBI350-0 and *m*-PBI splintered within an hour and fully dissolved by the end of the test (Figure 4A). The *m*-PBI350-0.25 was dissolved partially, rendering it permanently morphed. Notably, *m*-PBI350-2 did not exhibit solubility in DMAc. In PA, none of the samples dissolved after 1 h; however, a majority of them dissolved by the end of the test (Figure 4B). The *m*-PBI350-0 dissolved faster than *m*-PBI, which was likely due to its porosity, which led to greater contact with the solvent. The *m*-PBI350-0.25 turned into a gelatinous goop and did not retain any of its original shape. Even *m*-PBI350-2 exhibited some dissolution with slight changes in weight and discoloration of the solution. Generally, the *m*-PBI350-2 sample exhibited exceptional resistance to solvents, while the lesser cured membranes exhibited improved resistance compared to the non-cured *m*-PBI, thereby indicating successful cross linkage.

The doping levels and swelling ratio of the membranes were evaluated and are presented in Table 1. Controlling the swelling ratio is essential because a higher membrane thickness directly leads to increased ohmic resistance in the cell. The thermal treatment of membranes drastically suppresses their swelling behavior. For the uncured phase-inversed membrane (*m*-PBI350-0), extreme swelling was observed with swelling in the planar direction and an increased thickness more than twice the original value; however, these changes in the other samples were negligible or absent. For *m*-PBI350-2 and *m*-PBI350-3, prolonged thermal treatment gradually decreased the swelling ratio of the membranes until it stabilized. *m*-PBI350-1, *m*-PBI30-2, and *m*-PBI350-3 showed swelling ratio lower than that of m-PBI. However, semi-brittleness was also noted for *m*-PBI350-3 and above.

For all porous samples, the ADL of the membranes is higher than that of *m*-PBI, which is comparable to the ADL of direct synthesized samples [22,23,50]. Even with decreased doping levels, the ADL of the treated samples still exceeds that of normal *m*-PBI. Longer thermal treatment resulted in reduced acid uptake. Thus, it can be assumed that an increased degree of cross-linking leads to a more densely packed polymer matrix, thereby suppressing membrane swelling and reducing maximum acid uptake. Thus, the curing time should be optimized such that maximum performance is achieved.

**Figure 5:**
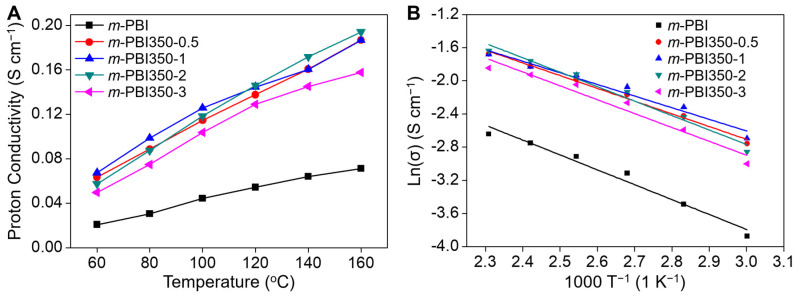
(**A**) Proton conductivity with respect to temperature and (**B**) Arrhenius plot for the proton conductivity of the phosphoric acid-doped membranes.

Proton conductivity is one of the key parameters in determining the performance of proton exchange membranes. Figure 5A depicts the proton conductivity of the samples with respect to time. The transport mechanism within the membrane is attributed to the Grotthus mechanism (i.e., protons hop through the network of hydrogen bonds) and vehicle mechanism (i.e., protons bound to the acid molecule move through the membrane) [51,52]. The porous nature of the membranes significantly increases the amount of free acid for operation in the system and improves proton conductivity. The *m*-PBI350-0 and *m*-PBI350-0.25 were not tested due to their instability at elevated temperatures. All the samples with longer treatment times than *m*-PBI350-0.5 exhibited excellent proton conductivities, as compared to *m*-PBI. A majority of the samples exhibit high proton conductivities with minimal differences; *m*-PBI350-2 achieved a conductivity of 0.194 S cm^−1^ at 160 °C. Above a certain doping level, it can be assumed that the amount of free acid in the matrix has little impact in further increasing the proton conductivity of the membranes. However, the proton conductivity of *m*-PBI350-3 is lower; this could be attributed to its lower acid retention ability and brittleness, which can cause a loss of acid during stabilization.

From Figure 5A, it can be seen that the conductivity of the membranes increases with temperature. Hence, an increase in temperature enables more favorable dynamics for both proton transfer and structural reorganization, thereby increasing the performance of the membranes, as reflected in Figure 5B. Considering this, their activation energy can be calculated using the Arrhenius equation (Equation (7)). Figure 5B presents typical Arrhenius plots obtained by plotting the proton conductivity of different membranes against the inverse of absolute temperature. The activation energy of the samples, which lies between 11.6 kJ mol^−1^ and 14.5 kJ mol^−1^, were calculated using the slopes of these plots. The data fit well with the theoretical values, suggesting that Grotthus mechanism is the primary pathway for the proton conduction process. The lower activation energy can be attributed to the presence of abundant free PA species in the membrane and their involvement in the transport process, as confirmed by the vast amount of free acid in the porous polymer matrix. Moreover, due to the porous morphology of the membrane, protons may move more easily, resulting in lower conduction activation energy [18].

Unlike Nafion^®^-based systems, which require external humidification, anhydrous fuel cell systems using PA as the medium cannot reobtain the acid once it leaches out. Furthermore, under prolonged operations, PA losses may occur due to the formation of water at the cathode end. Thus, PA retention ability is vital in maintaining the performance of PEM.

**Figure 6:**
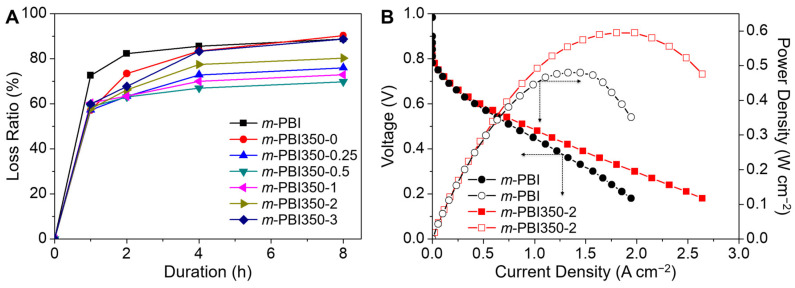
(**A**) Phosphoric acid retention of the membranes with respect to time. (**B**) Fuel cell performances in terms of power density and voltage with respect to the current density of *m*-PBI and *m*-PBI350-2.

Acid retention was tested to observe the effect of the porous structure of the membrane on its acid-trapping ability. The samples were subjected to water vapor at 100 °C, where water was allowed to coalesce and drop down on the surface of the membrane. During the course of the testing, the PA doping level for all membranes decreased constantly. Through testing for an extended period of time (~48 h), PA was completely removed from the membranes. The obtained acid loss ratios are presented in Figure 6A. It was observed that all porous *m*-PBI samples exhibit delayed acid losses. However, for *m*-PBI350-0 and *m*-PBI350-0.25, the loss ratio converged to that of *m*-PBI after 8 h of testing. For higher cross-linked samples, the loss ratio was lower than that of *m*-PBI, indicating that a higher degree of cross-linking leads to a tighter binding of PA on the membrane. Furthermore, a curing time of 0.5–2 h yields the highest retention ability. Escorihuela et al. [53] have previously demonstrated that PA leaching is prominent after a few cycles, thus a similar approach was employed to observe PA leaching behavior. The membrane was subjected to few cycles where the temperature was raised from 20 °C to 160 °C (first ramp) and 160 °C to 20 °C (second ramp) and repeated once more (Figure S1). The trend was the same as reported previously, wherein the first ramp showed high conductivity even at a lower temperature and the results after the first ramp show better correlation with temperature. This is possibly due to existence of water in the membrane and excess acid at the surface, which can contribute to proton conductivity but is quickly lost upon heating. The loss rate of *m*-PBI350-2 was lesser than that of *m*-PBI, but decreased nonetheless.

**Figure 7:**
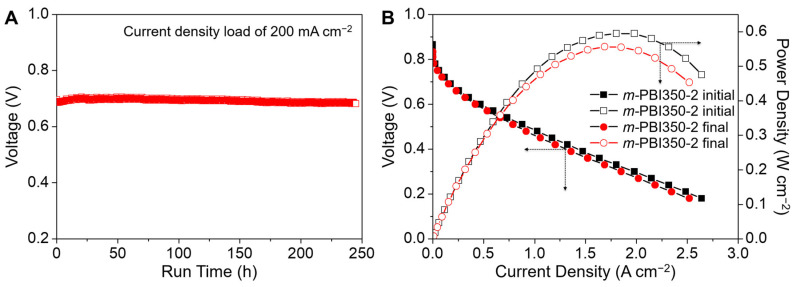
(**A**) Fuel cell performance of *m*-PBI350-2 with respect to time at a constant current density. (**B**) Cell performances before and after the durability test.

The polarization curves were measured using a single cell test at 160 °C with H_2_ and O_2_, without any external humidification (Figure 6B). The test was conducted using the *m*-PBI350-2 membrane, which exhibited the best performance in terms of proton conductivity and swelling behavior. The denser layer faced the cathode side, though its effect was not significant in previous study [54]. Low open circuit voltages are observed for *m*-PBI350-2 [55]. In addition, compared to *m*-PBI, *m*-PBI350-2 exhibited better performance with a maximum power density of 595 mW cm^−2^, even when considering membrane thickness. It also exhibited a stable performance without any apparent decline, which is commonly observed for damaged membranes with high cross-over [5,8,9], indicating the asymmetric nature of the membrane surface and the sufficient pores filled with liquid PA to warrant minimal gas cross-over (Figure 7A).

As *m*-PBI350-2 achieved a stable performance, the durability of the membrane was also tested (Figure 7B). The long-term durability loss of *m*-PBI is attributed to acid leaching [56], which is more extensive for a porous membrane than dense *m*-PBI because there is an excessive amount of free acid. The cell was placed under a current load of 200 mA cm^−2^, and a full polarization curve was obtained every 4 h. Moreover, the voltage was monitored constantly. After a run time of ~250 h, the cell voltage decreased from 0.705 V to 0.686 V when operated under 200 mA cm^−2^ (loss rate: 77.5 μV h^−1^). Although porous membranes feature lower durability than dense *m*-PBI [55], the leaching of free acid was minimized, similar to the acid retention ability.

## 4. Conclusions

Highly porous *m*-PBI membranes were successfully synthesized via phase inversion and employed in the HT-PEMFC environment using thermal cross-linking. The completion of the synthesis was confirmed based on FT-IR, TGA, and SEM. The porous *m*-PBI membranes were subjected to different thermal treatment conditions, yielding varying performances. SEM was used to investigate the porous nature and effect of thermal cross-linking on the morphology of the membranes. The amount of PA uptake was calculated, indicating improvements in swelling ratio and retention ability; however, the doping level was found to decrease upon an increase in the thermal treatment. The proton conductivities of *m*-PBI350-0.5 and membranes with longer treatment times increased; the maximum conductivity (0.194 S cm^−1^) was obtained using *m*-PBI350-2 and the minimum conductivity was 0.158 S cm^−1^, both of which are higher than that of *m*-PBI. The maximum power density was noted to be 595 mW cm^−2^ for *m*-PBI350-2. The results of *m*-PBI350-3 suggest that prolonged heat treatment can result in performance loss, whereas those of *m*-PBI350-0.25 indicated the necessity for sufficient cross-linking in HT-PEMFCs. Thus, it is important to optimize the curing time such that a maximum performance balance is achieved. The improved performance, compared to neat *m*-PBI, suggests that the proposed membranes are suitable electrolytes for HT-PEMFC systems. Moreover, future research is required for the optimization of pore size control in order to potentially improve membrane performance.

## Figures and Tables

**Table 1:** Swelling ratio and PA doping level of *m*-PBI350-x membranes.

Sample	Initial Thickness (μm)	Doped Thickness (μm)	Swelling Ratio (%)	Acid Doping Level (ADL)
*m*-PBI350-0	65	155	241	51.5
*m*-PBI350-0.25	64	113	77.2	50.4
*m*-PBI350-0.5	63	101	64.32	44.7
*m*-PBI350-1	62	91	43.5	38.0
*m*-PBI350-2	62	86	38.7	33.8
*m*-PBI350-3	62	85	37.1	29.9
*m*-PBI	49	72	50.6	11.0

## Supplementary Materials

The following are available online at https://www.mdpi.com/2073-4360/12/7/1604/s1, Figure S1: Proton conductivity of the m-PBI350-2 membranes during four consecutive temperature cycles, Table S1: Comparison of proton conductivity of PBI based membranes.
