# Supplementary material for: Phase Inversion-Induced Porous Polybenzimidazole Fuel Cell Membranes: An Efficient Architecture for High-Temperature Water-Free Proton Transport
Source: Polymers (Basel). 2020 Jul 19;12(7):1604. doi: 10.3390/polym12071604 (PMC7407769; doi:10.3390/polym12071604)
Supplement: Supplementary file 1 [file polymers-12-01604-s001.pdf]

Supplementary Information

Phase Inversion-Induced Porous Polybenzimidazole Fuel Cell Membranes: An Efficient Architecture for High-Temperature Water-Free Proton Transport

*Sangrae Lee‡, Ki-Ho Nam‡, Kwangwon Seo, Gunhwi Kim, and Haksoo Han**

Department of Chemical and Biomolecular Engineering, Yonsei University, 50 Yonsei-ro, Seodaemun-gu, Seoul 120-749, Republic of Korea

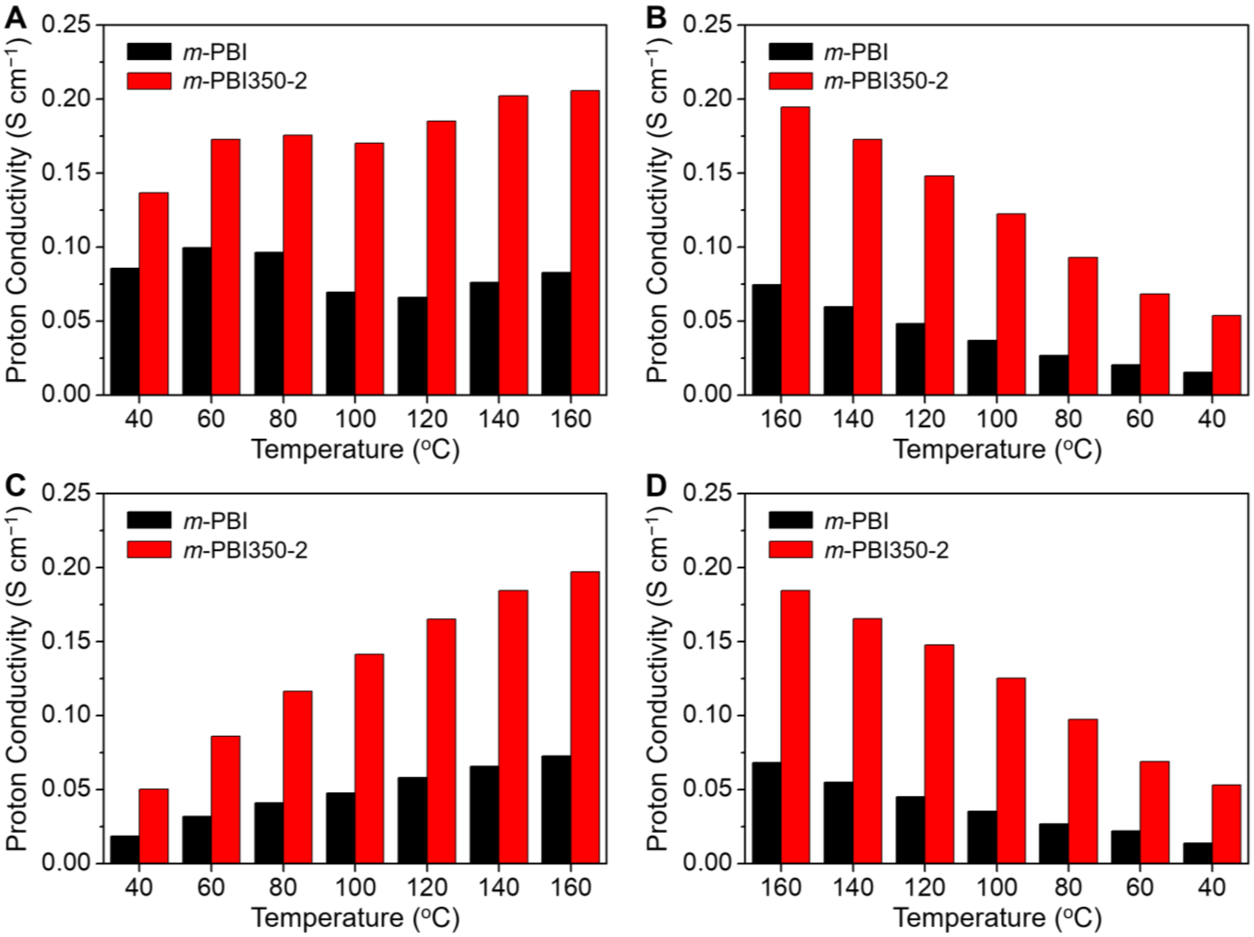

Figure S1. Proton conductivity of the *m*-PBI350-2 membranes during four consecutive temperature cycles.

Table S1. Comparison of proton conductivity of PBI based membranes

Ref	Membrane	Proton conductivity (mS cm^{-1})	Temperature (°C)	RH (%)
Int. J. Hydrogen Energy, **42**, 2636 (2017)	PBI/GO 2 wt%	129.7	165	Anhydrous
J. Power Sources, **401**, 149 (2018)	PBI/$CeGdP_2O_7$/GO 32 wt.%	199	180	5
J. Mater. Chem., **21**, 7480 (2011)	PBI/PBI-MWCNT 0.2 wt.%	85	160	Anhydrous
J. Mater. Chem., **21**, 7223 (2011)	PBI/phosphonated CNT 2.5 wt.%	~120	160	Anhydrous
Nanomaterials, **8**, 775 (2018)	PBI/ZIF-mix	91	200	Anhydrous
J. Power Sources, **276**, 62 (2015)	PBI/$BaZrO_3$ 4 wt.%	125	180	5
J. Mem. Sci., **383**, 78 (2011)	PBI/Clay 15 wt.%	120	150	12
J. Mater. Chem., **21**, 14897 (2011)	*o*-PBI/amine modified Si 20 wt.%	125	160	Anhydrous
**This work**	**PBI sponge**	**194**	**160**	**Anhydrous**

References

1. Uregen, N.; Pehlivanoglu, K.; Ozdemir, Y.; Devrim, Y. Development of polybenzimidazole/graphene oxide composite membranes for high temperature PEM fuel cells, *Int. J. Hydrogen Energy* **2017**, 42, 2636–2647. http://doi.org/10.1016/j.ijhydene.2016.07.009

2. Singh, B.; Devi, N.; Srivastava, A. K.; Singh, R. K.; Song, S.-J.; Krishnan, N.N.; Konovalova, A.; Henkensmeier, D. High temperature polymer electrolyte membrane fuel cells with Polybenzimidazole-Ce0.9Gd0.1P2O7 and polybenzimidazole-Ce0.9Gd0.1P2O7-graphite oxide composite electrolytes, *J. Power Sources* **2018**, 401, 149–157. https://doi.org/10.1016/j.jpowsour.2018.08.076

3. Suryani; Chang, C.-M.; Liu, Y.-L.; Lee, Y. M. Polybenzimidazole membranes modified with polyelectrolyte-functionalized multiwalled carbon nanotubes for proton exchange membrane fuel cells, *J. Mater. Chem.* **2011**, 21, 7480–7486. https://doi.org/10.1039/c1jm10439j

4. Kannan, R.; Kagalwala, H. N.; Chaudhari, H. D.; Kharul, U. K.; Kurungot, S.; Pilai, V. K. Improved performance of phosphonated carbon nanotube–polybenzimidazole composite membranes in proton exchange membrane fuel cells, *J. Mater. Chem.* **2011**, 21, 7223–7231. https://doi.org/10.1039/c0jm04265j

5. Escorihuela, J.; Sahuquillo, O.; Garcia-Bernabe, A.; Gimenez, E.; Compan, V. Phosphoric Acid Doped Polybenzimidazole (PBI)/Zeolitic Imidazolate Framework Composite Membranes with Significantly Enhanced Proton Conductivity under Low Humidity Conditions, *Nanomaterials* **2018**, 8, 775. https://doi.org/10.3390/nano8100775

6. Hooshyari, K.; Javanbakht, M.; Shabanikia, A.; Enhessari, M. Fabrication BaZrO3/PBI-based nanocomposite as a new proton conducting membrane for high temperature proton exchange membrane fuel cells, *J. Power Sources* **2015**, 276, 62–72. http://doi.org/10.1016/j.jpowsour.2014.11.083

7. Plackett, D.; Siu, A.; Li, Q.; Pan, C.; Jensen, J. O.; Nielson, S. F.; Permyakova, A. A.; Bjerrum, N. J. High-temperature proton exchange membranes based on polybenzimidazole and clay composites for fuel cells, *J. Mem. Sci.* **2011**, 383, 78-87. https://doi.org/10.1016/j.memsci.2011.08.038

8. Ghosh, S.; Maity, S.; Jana, T. Polybenzimidazole/silica nanocomposites: Organic-inorganic hybrid membranes for PEM fuel cell, *J. Mater. Chem.* **2011**, 21, 14897–14906. https://doi.org/10.1039/c1jm12169c
